# The illusion of empowerment: commercializing women’s health in the digital age

**DOI:** 10.1093/heapro/daag074

**Published:** 2026-05-29

**Authors:** Brooke Nickel, Tessa Copp, Barbara Mintzes

**Affiliations:** Sydney School of Public Health, Faculty of Medicine and Health, The University of Sydney, Edward Ford Building (A27), Sydney, NSW 2006, Australia; Sydney School of Public Health, Faculty of Medicine and Health, The University of Sydney, Edward Ford Building (A27), Sydney, NSW 2006, Australia; School of Pharmacy, Faculty of Medicine and Health, Susan Wakil Health Building (D18), The University of Sydney, Sydney, NSW 2006, Australia

Women’s health has long been under-researched, under-funded, and structurally marginalized (Sugimoto *et al.* 2019, Regensteiner *et al.* 2025, World Health Organisation 2026). From systematic exclusion from clinical trials to persistent diagnostic delays in conditions such as heart disease (Mikhail 2005, Nadarajah *et al.* 2023) and endometriosis (Harrilal-Maharaj 2025), the knowledge gaps are well documented. However, alongside the advancement of the digital age, rapidly expanding commercial markets promise to close these gaps not through enhanced evidence and structural reform, but through products and services (McCarthy *et al*. 2023, Brassart Olsen 2024, Wood *et al.* 2025).

Direct-to-consumer marketing and digital technologies (‘FemTech’) have been framed as a revolution in women’s autonomy, with women encouraged to track, test, optimize, and take control of their health through various interventions (Polis *et al.* 2026). Yet what is presented as empowerment may also represent a privatized response driving a market that monetizes unmet need while shifting responsibility from health systems to the individual woman (Copp *et al.* 2024, Berry 2025, Mintzes 2025). The intention is not to dismiss innovation or question women’s autonomy, as many women report positive experiences with these products and services. Rather, the concern lies not with their existence, but with the ways in which they are being framed, positioned, and marketed to women. Here we present four case studies to show how unmet needs of women have become a commercial opportunity in the digital age.

## Data as empowerment: fertility and cycle tracking apps

The use of fertility and cycle tracking apps has exponentially increased since their release over a decade ago (Bull *et al.* 2019, Rampazzo *et al.* 2024). These apps are marketed as tools for women’s bodily literacy, promising insight into ovulation, cycle regularity, mood, and fertility windows to ‘know your body better’ and ‘take control of your reproductive health’ through data (Graham *et al.* 2026a). For many women, these tools do provide value as they can support knowledge of cycle length, conception and contraception planning, and awareness of signs and symptoms (Broad *et al.* 2022, Riley and Paskova 2022, Arbeena *et al.* 2025, O’Brien and Iriarte 2025). However, the empowerment narrative of these apps glosses over key limitations (Graham *et al.* 2026a), with studies finding the apps’ predictive algorithms are inaccurate for the majority of women, especially those with irregular or varied cycles (Earle *et al.* 2021, Worsfold *et al.* 2021, Patel *et al.* 2024). These ovulation predictions are probabilistic, not diagnostic, and provide a perception of precision that may generate misplaced confidence for women or unnecessary anxiety (Broad *et al.* 2022, Riley and Paskova 2022, Arbeena *et al.* 2025, O’Brien and Iriarte 2025).

More fundamentally, these apps reframe healthcare access as self-surveillance (Kreitmair 2024, Sheridan Clay *et al.* 2025). Responsibility for meeting women’s needs or identifying any underlying abnormality shifts from clinicians and systems to the individual woman, who must interpret their own charts, fluctuations, and risk scores. Again, for some women this can be empowering, e.g. if a woman enters her data correctly, and understands and uses the apps’ predictions as a guide to support identification of her physical signs of ovulation, or takes her tracked menstrual cycle history to her clinician to support diagnosis and treatment, but for others it may lead to further confusion or anxiety (Patel *et al.* 2024). Furthermore, women’s individual reproductive data becomes a commercial asset. Even when privacy rules are technically followed, there is still an imbalance for women as they share their intimate health data (Levy and Romo-Avilés 2019, Patel *et al.* 2024, Mohan and Jenkins 2025), while companies decide how to use it and profit from it.

## Diagnostic consumerism: direct-to-consumer hormone testing

If apps offer women the chance to observe their bodies, direct-to-consumer tests offer the ability for women to diagnose themselves (Gram *et al.* 2024). While the emergence of direct-to-consumer testing is not particularly new, companies are now selling at-home panels to women measuring cortisol, oestrogen, progesterone, and other markers (Shih *et al.* 2023, Christakis *et al.* 2025), often under the broad banner of identifying ‘hormone imbalance’ (Ironmonger 2023, Willis 2025). The concept is compelling as relatively normal changes and symptoms for women such as fatigue, weight fluctuation, low mood, or irregular cycles may often feel overlooked and be dismissed in traditional consultations. Therefore, an easy-to-use test that promises objective answers appears validating and may offer acknowledgment for women (Ayala-Lopez and Nichols 2020).

Yet the clinical utility and accuracy of many of these panels are uncertain outside specific, well-defined contexts (Ho and Quick 2018, Galior and Baumann 2020, Gram *et al.* 2024). Hormones fluctuate across a women’s menstrual cycle and even throughout the day making a single-point measurements variable and difficult to interpret. Furthermore, what might be ‘balanced’ for one woman, could be different for another. Therefore, the idea that a woman’s hormones are ‘imbalanced’ is just a marketing term rather than a precise diagnosis leading the results to be overinterpretation of normal physiological variation. This can then lead to unnecessary anxiety and a cascade of unnecessary interventions for women including supplementation, repeat testing, or consultations leading to overdiagnosis and/or overuse (Moynihan *et al.* 2012, Brownlee *et al.* 2017), with many of these interventions offered by the same companies selling the tests (Nickel *et al.* 2025a, 2025b, Gram *et al.* 2026).

## Online hype and unsubstantiated claims: monetizing menopause products and services

The menopause market is also booming (Marsh 2026). This includes both the growth of menopause-focused products and menopause-specific clinics. The online marketing behind these so-called interventions often portray a broad range of symptoms as being linked to menopause, including not only hot flushes (vasomotor symptoms) and vaginal dryness but forgetfulness, joint aches and pains, anxiety, and depression. The implication is that hormone treatments are effective for these symptoms, with individually compounded bioidentical hormones often promoted, despite a lack of evidence of greater effectiveness or safety than synthetic hormones and less controls over product quality (Santoro *et al.* 2016).

Growing concern about the quality of care, lack of clinical guidance, and inconsistent access to evidence-based treatments, combined with a surge in products marketed to women, prompted an Australian parliamentary inquiry into menopause in 2024 (Randle *et al.* 2024, Carbone 2025). These products, including herbal tablets and patches, teas, powdered drink supplements, herbal enemas, and cooling mists as well as hormonal supplements, offer women tailored solutions to, often mild and/or normal, concerns and symptoms with no evidence to support their use. This intense marketing does not only target women’s vulnerability at a time of midlife change but also creates the impression that ‘untreated’ menopause is a wholly negative experience that is strongly associated with future ill-health (Wood *et al.* 2025).

Unsubstantiated claims that menopause is linked to cognitive decline, brain fog, and higher risks of dementia or heart disease can easily be understood to suggest that hormone treatment is needed to mitigate these potential risks. In contrast, a systematic review of international menopause guidelines found that none of at least moderate quality listed cognitive symptoms or dementia prevention as an indication for hormone treatment (Hemachandra *et al.* 2024). Hormone companies are therefore not allowed to promote their products for these uses. If a social media post is funded by a hormone companies, it also cannot legally promote these treatments for unapproved uses. However, these posts are not actively monitored by regulatory agencies and financial links to manufacturers, if present, may not be obvious. An analysis of Instagram menopause-related posts found that nearly half were advertisements (Arseneau *et al.* 2021). The top four categories of advertisements were nondrug treatments, clinician services, fitness/life coaching, and pharmaceuticals. Moreover, framing menopause as a new frontier of innovation risks obscuring structural causes of neglect (Thomas *et al.* 2024). The narrative here becomes one of disruption and entrepreneurial rescue, rather than of evidence-based solutions, sustained public investment, and health care workforce training around menopause (The Lancet 2024).

## Reproductive timing as optimization: elective egg freezing

Elective egg freezing extends the health empowerment narrative into life planning. Clinics and corporate benefit packages promote the technology as a way for women to take control of your reproductive timeline (Bayefsky *et al.* 2020, Beilby *et al.* 2020, Hammarberg *et al.* 2025). In an era of delayed partnership and career precarity, the appeal is understandable. While this intervention can indeed expand reproductive options for some women, it is expensive, physically demanding, and far from guarantees future live birth. Evidence to date shows that only 11% of women return to use their frozen eggs, and just 28% of those women achieve a live birth (Hirsch *et al.* 2024). Success rates vary with age at freezing and number of eggs retrieved, and marketing around egg freezing often highlights possibility more than the actual probability with success rates not clearly disclosed or masked by the overemphasis of the benefits (Beilby *et al.* 2020, Gürtin and Tiemann 2021).

Egg freezing also reframes structural inequality as an individual biological challenge. Instead of transforming societal and workplace cultures that discourage pregnancy or caregiving, the burden shifts to women to preserve their fertility proactively (Lacy-Nichols *et al.* 2022). While corporate sponsorship of egg freezing can signal support for choice, it also normalizes delayed childbearing in environments resistant to accommodating parental responsibilities. Here empowerment is entwined with biocapitalism, with reproductive potential becoming something to bank, store, and hedge, and the structural conditions that make women delay having babies when they are biologically better able remain largely unaddressed (van de Wiel 2022, Copp *et al.* 2024, McGrew and Rodgers 2025).

### The amplification of empowerment through social media

The rise of social media for health (Powell and Pring 2024) has significantly intensified both the spread and the persuasive power of women’s health empowerment narratives, allowing commercial interventions to be delivered directly and continuously to women through highly targeted advertising and influencer-led promotions (Nickel *et al.* 2025a, 2025b). Algorithmic systems have the ability to curate content based on intimate data about women’s bodies, emotions, and life stages including fertility, pregnancy, or menopause and create a personalized stream of messages that frame commercial products and digital tools and services as timely, necessary, and even morally responsible choices (John *et al.* 2025). Influencers and brand partnerships further blur the boundary between peer support, health advice, and marketing, presenting sponsored content in an informal and relational style that can obscure commercial interests and amplify claims of empowerment, self-care, and personal optimization (Heiss *et al.* 2025).

The social media environment therefore increases the risk that women are exposed to misleading or overstated health claims about effectiveness, safety, and clinical legitimacy. This is particularly evident when promotional content draws on anecdotal success stories (Richter *et al.* 2026) which can shape beliefs about treatment efficacy, treatment choices, and attitudes to health messages (Graham *et al.* 2026b). At the same time, regulatory frameworks have struggled to keep pace with the speed, scale, and global nature of digital marketing practices. Disclosure requirements, advertising standards, and health product regulations are often inconsistently applied or weakly enforced online, leaving significant gaps in oversight. As a result, responsibility for evaluating credibility and risk is shifted onto individual women, rather than being safeguarded through robust public regulation (Gram *et al.* 2025). This further entrenches a market-driven model of women’s health in which commercial narratives around empowerment circulate faster and more visibly than evidence-based information, reinforcing the normalization of consumer solutions while regulatory and institutional responses lag behind.

### Reclaiming true empowerment

Marketing of women’s health products and services with messages of empowerment are now commonly linked (Copp *et al.* 2024) . This is not to deny innovation or dismiss women’s agency as many women do report benefit from the products, tools and services being marketed (Grace *et al.* 2025). Again, the issue is not their existence, but how they are framed and marketed to women. When empowerment is equated with consumption and self-tracking and self-funding is considered autonomy, structural reform for women does not remain a priority and may be left behind or even get dismissed. Women also need access to accurate information on when medical care is or is not needed and on the balance of potential benefits and harms of interventions. This framing of empowerment as access to products and services also widens inequity as those women with resources are able to access enhanced versions of care, while others remain underserved. At the same time, political urgency to invest in robust, evidence-based women’s health services weakens as market-based solutions appear readily available, and ongoing gaps in public provision become easier to overlook.

True empowerment in women’s health would mean adequately funded research, equitable access to high-value care, accountability for data governance, transparent regulation of digital tools, and both top-down policy regulation and industry self-regulation of the misleading social media promotion of products and services. This would help to shift responsibility back towards systems rather than further onto women who are often already navigating complex biological and social pressures (Kickbusch and Holly 2023). While some digital innovations for women do hold promise, with vested interest driving investment in many of these and little structural commitment, the illusion of empowerment will continue to obscure evidence-based solutions and care for women. We must now begin to rebuild confidence in women’s health (Purnat *et al.* 2026) to improve trust and evidence-based care in the digital age for all women.

## Data Availability

No new data were generated or analysed in support of this research.
